# Hungarian Translation and Validation of the Pelvic Organ Prolapse Symptom Score

**DOI:** 10.1007/s00192-025-06090-5

**Published:** 2025-03-19

**Authors:** Szilard Kolumban, Nelli Farkas, Istvan Tiringer, Kalman Kovacs, Zoltan Nemeth, Balint Farkas

**Affiliations:** 1 Department of Obstetrics and Gynecology, University of Pecs School of Medicine, Pecs, Hungary; 2 Institute of Bioanalysis, Medical School, University of Pecs, Pecs, Hungary; 3 Department of Gynecology, Hospital St. John of God, Vienna, Austria; 4 Member of the HUN-REN–PTE Human Reproduction Research Group, Hungarian Academy of Sciences (MTA), Pecs, Hungary; 5 National Laboratory on Human Reproduction, University of Pecs, Pecs, Hungary; 6 Doctoral School of Health Sciences, Faculty of Health Sciences, University of Pecs, Pecs, Hungary; 7 Institute of Behavioural Sciences, Medical School, University of Pecs, Pecs, Hungary

**Keywords:** Adaptation, Hungarian, Pelvic organ prolapse symptoms, Quality of life, Screening questionnaire, Urogynecology

## Abstract

**Introduction and Hypothesis:**

Pelvic organ prolapse (POP) significantly affects women’s quality of life, making the availability of validated, culturally adapted tools for reliable symptom evaluation essential. In this study, the Pelvic Organ Prolapse Symptom Score (POP-SS) was translated into Hungarian and validated, with the assessment of its psychometric properties for clinical and research use.

**Methods:**

In total, 125 women diagnosed with symptomatic POP (Pelvic Organ Prolapse Quantification Score—POP-Q stage ≥ 2) completed the Hungarian POP-SS (POP-SS-H), the Hungarian version of the Australian Pelvic Floor Questionnaire (AFPQ-H), and the short version of the World Health Organization Quality of Life questionnaire (WHOQOL-BREF). Exploratory factor analysis was performed, McDonald’s *ω* was used to assess internal consistency, and intraclass correlation coefficients (ICCs) were used to examine test–retest reliability over a 2-week interval.

**Results:**

The POP-SS-H demonstrated strong psychometric properties (overall ICC = 0.96, *p* < 0.001). Item ICCs were 0.69–0.99, with “difficulty in emptying the bladder” being the most stable. The scale has a two-factor ("prolapse and urinary distress" and "abdominal strain and bowel discomfort”) structure. McDonald’s *ω* was 0.75, confirming its good internal consistency. The scale’s convergent validity was demonstrated through correlations with AFPQ-H and WHOQOL-BREF subscale scores.

**Conclusions:**

The POP-SS-H is a reliable and valid instrument for the assessment of prolapse-related symptoms. Its high degrees of test–retest reliability and internal consistency make it suitable for clinical follow-up and research. The tool addresses a significant gap in pelvic-floor disorder management in Hungary, although further research is recommended to assess its sensitivity in the tracking of symptom changes after treatment.

## Introduction

Anatomical and functional pelvic-floor changes (i.e., genital prolapse, and urinary and fecal incontinence) are taboo topics among affected women, even in current times. Between the ages of 40 and 85 years, 41–50% of women develop objectively detectable asymptomatic pelvic organ prolapse (POP) or urinary incontinence (UI), and 3% of patients report symptoms of these conditions [1]. By 2050, the number of women with POP is expected to increase by 46% to 4.9 million in the USA [2]. The real prevalence of these diseases, however, is difficult to evaluate, owing to the inconvenience of their symptoms and the lack of elderly patients’ compliance with gynecological consultation attendance. POP becomes more prevalent with advanced age, and conservative treatments alone often fail to achieve satisfactory symptom relief for patients [3].

With the onset of symptoms (pelvic pressure or heaviness, vaginal discomfort, bulging or protrusion, and urinary and bowel symptoms), patients’ quality of life, self-esteem, social relationships, sexual life, and body image begin to deteriorate. Because of the multifactorial nature of the symptoms, the following of patients can be challenging. It is not uncommon for patients to hold the misconception that POP is not a disease. Once treatment, whether conservative or surgical, has begun, the most reliable way to follow patients is with the use of validated questionnaires in addition to physical examination [4, 5]. The use of questionnaires that have not been validated in the population of interest may yield unreliable and incorrect results [6]; questionnaires that have been translated into the patients’ mother tongue and validated are required.

Hagen et al. [7, 8] developed the Pelvic Organ Prolapse Symptom Score (POP-SS) questionnaire for English-speaking women in 2000 and published on the psychometric properties of the tool in 2009 and 2010. The objective of this questionnaire is to facilitate patient comparison for the assessment of the impacts of different treatment modalities on prolapse-related symptoms. It can also be used for long-term follow-up. To date, the POP-SS tool has been successfully translated into Turkish [9], Amharic [10], Chinese [11], and Sidaamu Afoo [12], and these versions have been found to have acceptable psychometric properties. To our knowledge, however, no report describes the psychometric properties of a Hungarian version of this questionnaire.

The aims of this study were to translate the POP-SS questionnaire into Hungarian (POP-SS-H) and validate the translated version to ensure that it is culturally and linguistically appropriate for Hungarian-speaking women. Translation, back-translation, and psychometric evaluation were performed to assess the reliability, validity, and applicability of the POP-SS in the Hungarian context for effective patient follow-up and the comparison of prolapse-related symptoms. The secondary objective was to make the POP-SS-H publicly available for research and clinical purposes.

## Materials and Methods

### Translation and Linguistic Validation of the Pelvic Organ Prolapse Symptom Score Questionnaire

The original developer of the questionnaire (S. Hagen) approved the entire translation and validation process. Two physicians who were fluent in English and native speakers of Hungarian translated the original POP-SS instrument from English into Hungarian. A physician who was a native speaker of both languages independently performed back-translation, and the back-translated questionnaire was reviewed by the original author (S. Hagen). Linguistic validation was carried out according to published guidelines [13]. To develop the final POP-SS-H, face-to-face interviews were conducted with patients not included in the main study to identify any difficulty with the understanding and interpretation of the questions.

### Study Population and Data Collection

The number of participants was determined by a priori power analysis. Consecutive outpatients diagnosed with POP at a Clinical Center in an Obstetrics and Gynecology Department and a Private Clinic between June 2023 and August 2024 were included in the study. All patients provided informed consent to study participation, which was fully voluntary and anonymous. Eligible patients were aged > 18 years, able to speak and read Hungarian, and diagnosed with symptomatic POP (POP-Q ≥ 2). Pregnant women, those with chronic pelvic pain lasting for ≥ 6 months, and those with asymptomatic POP identified during routine gynecological examinations were excluded. The patients were interviewed in person. As a preliminary step, 20 patients participated in the evaluation of the test–retest reliability of the POP-SS-H over a 2-week period during which they received no treatment. They participated in interviews at baseline and 2 weeks with the administration of the same questions. Following the preliminary study, 125 outpatients were enrolled in the study.

### Pelvic Organ Prolapse Symptom Score Questionnaire

The original POP-SS questionnaire comprises seven questions. Respondents are asked to indicate the frequency with which they have experienced the following symptoms in the last 4 weeks: a feeling of something coming down from the vagina, an uncomfortable feeling in the vagina that worsens when standing, a feeling of heaviness in the lower abdomen, a dragging feeling in the lower back, difficulty in emptying the bladder, a feeling of incomplete bladder emptying, and a feeling of incomplete bowel emptying. Responses are structured by a five-point scale ranging from 0 to 4 (0 = symptom never felt, 1 = occasionally, 2 = sometimes, 3 = most of the time, and 4 = all the time). Higher scores reflect greater symptom severity. Total scores (0–28) are calculated by summing all question responses. Scores > 0 indicate the presence of symptoms associated with prolapse [7, 8].

Validation was conducted with the POP-SS questionnaire, the Hungarian version of the Australian Pelvic Floor Questionnaire (AFPQ-H) [14], and, with the permission of the World Health Organization (WHO), the short version of the World Health Organization Quality of Life questionnaire (WHOQOL-BREF) [15]. We also gathered sociodemographic data from the patients.

### Hungarian Version of the Australian Pelvic Floor Questionnaire

The AFPQ-H measures aspects of pelvic-floor dysfunction, including prolapse-related symptoms, bowel and urinary distress, and sexual function, similar to the POP-SS questionnaire. It has four domains: prolapse, bowel symptoms, bladder symptoms, and sexual function. Most item responses are structured by a four-point scale, with exceptions for questions addressing defecation frequency, bowel consistency, sufficient lubrication, and the reason for sexual abstinence. The scores for each relevant question are divided by the total score for the corresponding domain and multiplied by 10, resulting in domain scores ranging from 0 to 10. These domain scores are summed to derive a global pelvic-floor dysfunction score, with a maximum value of 40. Higher scores indicate more severe pelvic-floor dysfunction [14].

### Short Version of the World Health Organization Quality of Life Questionnaire

The WHOQOL-BREF is a widely used questionnaire designed to assess individuals’ perceived quality of life across four key domains: physical health (7 items), psychological health (6 items), social relationships (3 items), and environmental health (8 items); it also includes one item each on general quality of life and perceived health. Each individual item of the WHOQOL-BREF is scored from 1 to 5 on a response scale, which is stipulated as a five-point ordinal scale. The scores are then transformed linearly to a 0–100 scale. The WHOQOL-BREF maintains strong psychometric properties while offering a more practical and time-efficient alternative to the full-length version for use in clinical and research settings. The questionnaire has been validated in diverse cultural and clinical populations, making it a reliable tool for cross-cultural comparisons and a valuable instrument for the assessment of the quality of life of healthy individuals and those with various health conditions. Its brevity and comprehensive nature enable its frequent use in the monitoring of treatment outcomes and to guide clinical decision making [16].

### Statistical Analysis

All statistical analyses were performed with the R-based JAMOVI 2.3.28 software [17]. The normal distribution of the variables was checked by the Kolmogorov–Smirnov test. As the assumption of normal distribution was not met for the scales and subscales analyzed, the relationships between them were examined using nonparametric Spearman’s correlation analysis.

### Validation

#### Evaluation of Construct Validity

Principal component analysis (PCA) with Promax rotation was carried out to analyze the structure of the POP-SS items. The suitability of the items of the POP-SS for factor analysis was tested using Bartlett’s test of sphericity and the Kaiser–Meyer–Olkin measure of sampling adequacy. The number of components was determined by parallel analysis and the Kaiser criterion (eigenvalue > 1) [18].

#### Evaluation of Reliability

To assess the internal consistency of the questionnaire, McDonald’s *ω* was used. This statistic is a more accurate measure of reliability than Cronbach’s *α* when the precondition of the tau equivalent model is not met, i.e., the data are not normally distributed [19]. *ω* values between 0.7 and 0.8 are acceptable.

The final POP-SS-H questionnaire was administered to 20 women attending our outpatient clinic. Two weeks later, the questionnaire was re-administered to test its temporal stability. Test–retest reliability of the items and the hole scale were assessed by calculating the intraclass correlation (two-way mixed effects, consistency, single rater). The patients received no conservative or surgical treatment during this period.

#### Evaluation of Convergent Validity

The Hungarian Australian Pelvic Floor Questionnaire (APFQ-H) questionnaire [14] was used for the assessment of convergent validity, given that it has been used previously to assess the impacts of POP, UI, fecal incontinence, and sexual problems on patients’ daily lives. Convergent validity was characterized by Spearman correlation coefficients between subscales. A rho value of 0.6–0.8 indicates a strong relationship between subscales, values of 0.4–0.6 a moderate relationship, and values of 0.2–0.4 a weak relationship.

## Results

During the initial face-to-face interviews with patients no difficulties were found.

### Sociodemographic Characteristics

The sociodemographic characteristics of the study group are summarized in Table 1. The mean age ± standard deviation (SD) of the 125 participants was 63.87 ± 13.04 (range, 31–91) years. The largest proportion (40.8%) of participants lived in county seats; 32.8% resided in other cities, 25.6% lived in villages, and 0.8% lived in rural areas. The majority (56%) of participants were married; 26.4% were widowed, 9.6% were divorced, 7.2% were in relationships but unmarried, and 0.8% were single. The largest proportion (40.8%) of participants had completed secondary education; 25.6% held higher education degrees, 24% had vocational training, and 8.8% had elementary-school educational levels. More than half (54.4%) of the participants were retired; 31.2% worked full time, and smaller percentages worked part time or were unemployed.

**Table 1:** Demographic characteristics of the study participants

Category	Variable	Data	Count (*n*)
Age, mean (SD)		63.87 (13.04)	125
Residence (%)	County seat	40.8	51
	Other city	32.8	41
	Village	25.6	32
	Rural area	0.8	1
Marital status (%)	Married	56.0	70
	Widowed	26.4	33
	Divorced	9.6	12
	In a relationship	7.2	9
	Single	0.8	1
Education level (%)	Secondary education	40.8	51
	Higher education	25.6	31
	Vocational training	24.0	30
	Elementary school	8.8	11
	Less than elementary	0.8	1
Employment status (%)	Retired	54.5	68
	Full-time employment	31.2	39
	Part-time employment	0.8	1
	Unemployed	8.0	10

### Descriptive Statistics for the Hungarian Pelvic Organ Prolapse Symptom Score Questionnaire

The minimum score was 0, suggesting that some participants experienced no symptoms, and the maximum score was 28.0, representing severe prolapse-related symptoms. Scores were ≤ 7 for 25% of the participants, ≤ 12 for 50%, and ≤ 16.0 for 75% of participants. The POP-SS distribution was close to normal (*p* = 0.042, Kolmogorov–Smirnov test).

### Construct Validity

Bartlett’s test of sphericity (*χ*^2^ = 262, *p* < 0.001) and the Kaiser–Meyer–Olkin measure of sampling adequacy (KMO = 0.66) showed that the items of the POP-SS-H instrument had adequate common variance for factor analysis. PCA led to the extraction of two components based on parallel analysis and the Kaiser criterion (eigenvalue > 1). The factors were rotated using a Promax approach because they were assumed to be correlated. The two components explain 60% of the item variance. Four items (“feeling of something coming down from the vagina,” “an uncomfortable feeling or pain in your vagina,” “difficulty in emptying the bladder,” and “a feeling of incomplete bladder emptying”) loaded on factor 1 (named “prolapse and related urinary symptoms”), with factor loadings ranging from 0.54 to 0.94. Three items (“a feeling of heaviness in the lower abdomen,” “a dragging feeling in the lower back,” and “a feeling of incomplete bowel emptying”) loaded on factor 2 (named “abdominal and bowel discomfort”), with factor loadings ranging from 0.59 to 0.86 (Table 2). Components 1 and 2 explained 33.2% and 26.4% (total, 50.6%) of the common variance respectively (eigenvalues = 2.8 and 1.4 respectively). The items showed adequate communality (0.36–0.82; Table 2).

**Table 2:** Exploratory factor analysis results of Hungarian Pelvic Organ Prolapse Symptom Score (POP-SS-H)^a^

POP-SS-H	Component loadings		Communality
	Component 1	Component 2	
Item 1	0.544		0.38
Item 2	0.585		0.573
Item 3		0.862	0.736
Item 4		0.821	0.58
Item 5	0.906		0.73
Item 6	0.936		0.816
Item 7		0.585	0.361
	33.2%^b^	24.6%^b^	

^a^Principal component analysis was performed with Promax rotation. Loadings < 0.35 are suppressed

^b^Explained variance

### Reliability

The scale showed acceptable internal consistency (*ω* = 0.75; Table 3). Some items fit the scale well, others fit the scale acceptably (see Table 3 for how the *ω* value would change after deleting the given item). Intraclass correlation coefficients (ICCs) for test–retest reliability and the total POP-SS were between 0.69 and 0.99 (*p* < 0.001; Table 4).

**Table 3:** Internal consistency of the Hungarian Pelvic Organ Prolapse Symptom Score (POP-SS-H)

POP-SS-H	*ω* if item dropped
Item 1	0.73
Item 2	0.70
Item 3	0.73
Item 4	0.76
Item 5	0.71
Item 6	0.69
Item 7	0.75

**Table 4:** Test–retest reliability of item and total Hungarian Pelvic Organ Prolapse Symptom Score (POP-SS-H) scores

Variables	ICC^a^	95% CI	*p* value
POP-SS	0.96	0.90–0.98	< 0.001
POP1	0.91	0.79–0.96	< 0.001
POP2	0.73	0.43–0.88	< 0.001
POP3	0.69	0.36–0.86	< 0.001
POP4	0.91	0.78–0.96	< 0.001
POP5	0.99	0.97–1.00	< 0.001
POP6	0.90	0.77–0.96	< 0.001
POP7	0.91	0.80–0.97	< 0.001

*ICC* intraclass correlation coefficient, *CI* confidence interval

^a^Single-rating, two-way, mixed-effects model

### Convergent and Divergent Validity

The APFQ-H and WHOQOL-BREF questionnaires were used to test convergent and divergent validity. Although the POP-SS questionnaire is used as a consistent scale for screening, our factor analysis separated the POP-SS-H items into two components. The results also suggest that the first component might not be homogeneous, as the prolapse symptom items were related to it less strongly than were the insufficient bladder-emptying items. Thus, when examining convergent validity, we compared the prolapse symptoms (items 1 and 2) and bladder-emptying symptoms (items 5 and 6) subscale scores separately with the APFQ-H and WHOQOL-BREF subscale scores (Table 5).

**Table 5:** Convergent and divergent validity

	POP-SS (sum)	POP-SS prolapse^a^	POP-SS abdominal^b^	POP-SS bladder^c^
APFQ bladder (*n* = 123)	0.526***	0.362***	0.293***	0.489***
APFQ bowel (*n* = 123)	0.346***	0.225*	0.347***	0.241**
APFQ prolapse (*n* = 123)	0.624***	0.721***	0.322***	0.370***
APFQ sexual function (*n* = 52)	0.332*	0.189	0.399**	0.173

*APFQ* Australian Pelvic Floor Questionnaire, *POP-SS* Pelvic Organ Prolapse Symptom Score

*p < 0.05

**p < 0.01

***p < 0.001

^a^Items 1 and, 2

^b^Items 3, 4, and 7

^c^Items 5 and 6

The total POP-SS-H was related most closely to the APFQ-H prolapse (*ρ* = 0.624) and bladder (*ρ* = 0.526) symptoms subscales. POP-SS-H subscale scores also showed the strongest correlations with the corresponding AFPQ-H subscale scores for bladder symptoms (*ρ* = 0.489), rectal function (*ρ* = 0.347), and prolapse symptoms (*ρ* = 0.721).

The physical domain of the WHOQOL-BREF showed moderate to weak negative correlations with the total POP-SS-H and scores for items representing the main symptom clusters. The strongest association was with prolapse symptoms (ρ = –0.473). Associations with the WHOQOL-BREF psychological domain were somewhat weaker. Of the main symptom groups, the strongest association was with abdominal symptoms (*ρ* = −0.273; Table 6).

**Table 6:** Associations of POP-SS-H scores with the Short Version of the World Health Organization Quality of Life questionnaire (WHOQOL-BREF) psychological and physical domain scores

	POP-SS (sum)	POP-SS prolapse^a^	POP-SS abdominal^b^	POP-SS bladder^c^
WHOQOL-BREF physical (*n* = 123)	−0.499***	−0.473***	−0.364***	−0.298***
WHOQOL-BREF psychological (*n* = 123)	−0.271**	−0.145	−0.273**	−0.204*

*POP-SS* Pelvic Organ Prolapse Symptom Score

*p < 0.05

**p < 0.01

***p < 0.001

^a^Items 1 and, 2

^b^Items 3, 4, and 7

^c^Items 5 and 6

## Discussion

To our knowledge, this study is the first in which a Hungarian version of the POP-SS questionnaire has been validated. To date, our study group has translated a limited number of questionnaires in the field of urogynecology into Hungarian and validated them [14, 20, 21].

The validated POP-SS-H is an essential tool for the accurate assessment of POP symptoms among Hungarian-speaking women. The use of validated, culturally and linguistically adapted questionnaires is crucial for reliable symptom measurement and long-term follow-up in urogynecological practice. In line with other versions of the POP-SS instrument in other languages, such as Turkish, Amharic, and Chinese, the POP-SS-H has good psychometric properties, including internal consistency, test–retest reliability, and convergent and divergent validity.

The 125 participants in this study made up a predominantly older, retired population with a mean age of 63.87 years, which is within the age group most commonly affected by POP. The large numbers of married (56%) and widowed (26.4%) participants may have influenced symptom reporting, particularly in domains related to sexual function.

As POP encompasses multiple symptoms that are the consequences of pelvic-floor weakening and do not always occur simultaneously [16], the POP-SS-H does not function as a homogeneous scale. The factor structure obtained for the POP-SS questionnaire in this study differs from those obtained in several other studies [9–12]. The first component contains four items describing prolapse and bladder-emptying symptoms and was found to be related more closely to the latter than to the former. The second component contains items for abdominal discomfort symptoms and less closely related defecation problems. This bifurcation of symptom domains reflects the heterogeneity of pelvic-floor disorders. The differentiation between prolapse and bladder-emptying symptoms mirrors previous findings suggesting that pelvic-floor dysfunction might often encompass anatomical displacement and functional impairments, such as incomplete bladder emptying [22]. This difference in factor structure may be attributed to several factors specific to our study population, such as the inclusion of only patients with POP-Q ≥ 2 and potentially pronounced prolapse and bladder dysfunction symptom overlap. Although POP is often associated with urinary symptoms, the inclusion of patients with only UI may have led to the detection of a different factor structure.

The internal consistency of the POP-SS-H was confirmed with McDonald’s *ω*; although the scale items focused on different symptoms, the patients gave similar answers. The questionnaire also demonstrated test–retest reliability, with ICCs for the items and total score ranging from 0.69 to 0.99 (*p* < 0.001). The "difficulty in emptying the bladder" item demonstrated the most temporal stability (ICC = 0.99), suggesting that patients might consistently report this symptom over time. In contrast, the "feeling of incomplete bowel emptying" item had the lowest ICC (0.69), although still with acceptable reliability. The ICC for the total POP-SS was 0.96, confirming that the questionnaire as a whole is reliable for repeated use.

These results indicate that the POP-SS-H is a highly reliable tool for multiple assessments of POP symptoms. It demonstrated strong test–retest reliability, confirming that it retains temporal stability, which is crucial for clinical follow-up and research applications. Such stability is essential, for example, for the evaluation of the outcomes of conservative (e.g., pelvic-floor exercises, use of different types of pessaries) and surgical (e.g., prolapse-repair surgery) POP treatments. Other versions of the POP-SS questionnaire have demonstrated similar reliability metrics [9–12], further supporting the robustness of this instrument for the evaluation of prolapse-related symptoms in different populations. The convergent validity of the POP-SS-H was supported by strong–moderate correlations with the AFPQ-H prolapse and bladder subscales, strengthening its validity for the measurement of prolapse-related symptoms in the Hungarian population.

The clinical usefulness of the POP-SS-H for health care providers is twofold. First, it is a short, reliable questionnaire that can be readily integrated into daily practice. Second, it can be used to monitor the effectiveness of various treatment methods.

The validation of the POP-SS-H also underscores the broader implications of pelvic-floor disorders for women’s quality of life. POP significantly impairs women’s physical health, sexual function, and mental well-being. The associations of POP-SS-H scores with WHOQOL-BREF physical and psychological domain scores reinforce the notion that prolapse symptoms are not only physically disabling but also contribute to emotional distress and reduced self-esteem. The strongest association in this study was between the POP-SS-H prolapse symptoms score and the WHOQOL-BREF physical health domain score, indicating that physical well-being deteriorates markedly with increasing prolapse severity. This finding is consistent with previous reports of greater daily activity limitations leading to decreased physical function in women with severe prolapse [22, 23]. The weaker correlation with the psychological domain score suggests that prolapse symptoms, though primarily physical, do contribute to emotional stress, anxiety, and depression, especially when they are chronic or severe [23].

A major strength of this study is the rigorous methodological approach taken for the linguistic validation of the scale, which involved translation, back-translation, and patient interviews to ensure cultural and linguistic appropriateness. Additionally, a robust statistical analysis, including exploratory factor analysis and reliability testing, was performed to confirm the good psychometric properties of the POP-SS-H. The inclusion of convergent and divergent validity testing further strengthened confidence in the validity of this tool for the measurement of prolapse-related symptoms. Finally, the POP-SS-H is a valuable resource for health care providers in our country, as it is a reliable and accessible instrument for clinical practice and research applications.

The study also has some limitations to consider. The study population was drawn from two clinical centers, which may limit the generalizability of the findings to the wider Hungarian population. Additionally, although the questionnaire demonstrated good psychometric properties, the investigation of its sensitivity in detecting changes in symptom severity over time, especially after treatment, is warranted. Another limitation of this study is that we included only patients with symptomatic prolapse (POP-Q ≥ 2). Additionally, the severity of prolapse was not documented during the course of the study, which may have limited the ability to fully assess the relationship between symptom severity and prolapse grade.

## Conclusion

This study demonstrated that the POP-SS-H has good psychometric properties, confirming its reliability and validity as a tool for the assessment of prolapse-related symptoms in Hungarian-speaking women. The scale’s high test–retest reliability renders it suitable for the monitoring of symptom progression and treatment outcomes in urogynecological practice. The provision of this culturally and linguistically adapted instrument addresses a critical gap in the management of pelvic-floor disorders in Hungary. In future research, assessment of the sensitivity of the POP-SS-H in the post-treatment monitoring of changes in symptoms would be beneficial.

## Data Availability

The data that support the findings of this study are available from the corresponding author, [IT], upon reasonable request.
